# Supplementary material for: Membrane binding controls ordered self-assembly of animal septins
Source: eLife. 2021 Apr 13;10:e63349. doi: 10.7554/eLife.63349 (PMC8099429; doi:10.7554/eLife.63349)
Supplement: Transparent reporting form [file elife-63349-transrepform.pdf]

eLIFE
1st Floor
24 Hills Road
Cambridge CB2 1JP, UK
P 01223 855340
W elifesciences.org
T @elife

eLife's transparent reporting form

We encourage authors to provide detailed information *within their submission* to facilitate the interpretation and replication of experiments. If you have any questions, please contact us: editorial@elifesciences.org.

Sample-size estimation

- You should state whether an appropriate sample size was computed when the study was being designed
- You should state the statistical method of sample size computation and any required assumptions
- If no explicit power analysis was used, you should describe how you decided what sample (replicate) size (number) to use

Please outline where this information can be found within the submission (e.g., page numbers or figure legends), or explain why this information doesn't apply to your submission:

We do not use any explicit power analysis in this work. All experiments were undertaken with purified proteins and lipids, so we anticipated a narrow range of variations across replicates as compared to measurements on cells or organisms. The number of technical replicates was generally two or more, and we indeed found a high level of reproducibility for all the features analysed.

Replicates

- You should report how often each experiment was performed
- You should include a definition of biological versus technical replication
- The data obtained should be provided and sufficient information should be provided to indicate the number of independent biological and/or technical replicates
- If you encountered any outliers, you should describe how these were handled
- Criteria for exclusion/inclusion of data should be clearly stated
- High-throughput sequence data should be uploaded before submission, with a private link for reviewers provided (these are available from both GEO and ArrayExpress)

Please outline where this information can be found within the submission (e.g., page numbers or figure legends), or explain why this information doesn't apply to your submission:

eLife Sciences Publications, Ltd is a limited liability non-profit non-stock corporation incorporated in the State of Delaware, USA, with company number 5030732, and is registered in the UK with company number FC030576 and branch number BR015634 at the address 1st Floor, 24 Hills Road, Cambridge CB2 1JP | August 2014

- Biological replicates: Information on biological replicates is provided in the Materials and Methods section. Our entire study is based on measurements on biochemically reconstituted samples assembled from purified proteins and commercially available purified lipids. The majority of the experiments are performed with full-length fly septin hexamers, for which we performed a total of 10 protein preparations in three labs (Mavrakis, Koenderink, Bertin labs) and verified that the protein quality by SDS-PAGE and by TEM analysis was constant. QCM-D and AFM experiments were performed on 5 protein preps; TIRF on 3 preps; and TEM and STEM on 2 different preps. We prepared 2 different preps for C-terminally truncated fly septins, 1 prep for yeast septins, and 1 prep for mammalian septins.
- Technical replicates: For all quantitative measurements, the number of times each experiment was performed is provided in the main text where numbers (average±S.D.) are reported or in the figure captions: TIRF data (Fig. 2; Figure 2 – Figure supplement 1), AFM measurements (Fig 5), STEM measurements (Figure 1 – Figure supplement 2).
- Outliers: There are no outliers excluded.

Statistical reporting

- Statistical analysis methods should be described and justified
- Raw data should be presented in figures whenever informative to do so (typically when N per group is less than 10)
- For each experiment, you should identify the statistical tests used, exact values of N, definitions of center, methods of multiple test correction, and dispersion and precision measures (e.g., mean, median, SD, SEM, confidence intervals; and, for the major substantive results, a measure of effect size (e.g., Pearson's r, Cohen's d)
- Report exact p-values wherever possible alongside the summary statistics and 95% confidence intervals. These should be reported for all key questions and not only when the p-value is less than 0.05.

Please outline where this information can be found within the submission (e.g., page numbers or figure legends), or explain why this information doesn’t apply to your submission:

- We do not use explicit statistical analysis/statistical tests.
- Raw data are presented in figure for TIRF measurements (Fig. 2; Figure 2 – Figure supplement 1), QCM-D measurements (Figure 1 – Figure supplement 2), STEM measurements (Figure 1 – Figure supplement 2), and AFM measurements (Fig. 5).
- Values of *N* are specified in the figure captions, and where relevant, mean and S.D. are specified.

(For large datasets, or papers with a very large number of statistical tests, you may upload a single table file with tests, Ns, etc., with reference to page numbers in the manuscript.)

Additional data files ("source data")

- We encourage you to upload relevant additional data files, such as numerical data that are represented as a graph in a figure, or as a summary table
- Where provided, these should be in the most useful format, and they can be uploaded as "Source data" files linked to a main figure or table
- Include model definition files including the full list of parameters used
- Include code used for data analysis (e.g., R, MatLab)
- Avoid stating that data files are "available upon request"

Please indicate the figures or tables for which source data files have been provided:

n/a
